# Decreased brain-expressed X-linked 4 (BEX4) expression promotes growth of oral squamous cell carcinoma

**DOI:** 10.1186/s13046-016-0355-6

**Published:** 2016-06-13

**Authors:** Wei Gao, John Zeng-Hong Li, Si-Qi Chen, Chiao-Yun Chu, Jimmy Yu-Wai Chan, Thian-Sze Wong

**Affiliations:** Department of Surgery, The University of Hong Kong, Hong Kong SAR, China; Department of Otolaryngology, The First People’s Hospital of Foshan, Foshan, Guangdong Province China

**Keywords:** Oral cancer, Squamous cell carcinoma, Brain-expressed X-linked family, BEX4, Tumor growth, S100A family

## Abstract

**Background:**

Brain-expressed X-linked (BEX) 4 is a member of BEX family. The functional role of BEX4 in oral squamous cell carcinoma (OSCC) remains unknown.

**Methods:**

Expression level of BEX family members (BEX1-5) in OSCC tissues and the paired normal epithelial were examined. Functions of epigenetic changes (DNA methylation and histone modifications) on BEX4 suppression in OSCC were examined by zebularine and trichostatin A (TSA) treatment on OSCC cell lines. Lentivector containing full-length BEX4 was used to generate OSCC cell lines with stable BEX4 expression. Effects of BEX4 expression on OSCC proliferation were monitored with xCELLigence RTCA real-time cell analyzer. BEX4-overexpressing CAL27 was implanted into nude mice to evaluate the effects on tumor growth in vivo. The signaling pathways regulated by BEX4 in OSCC was explored using human whole-transcript expression microarray.

**Results:**

Among the 5 BEX family members, BEX1 and BEX4 showed significant down-regulation in OSCC (*P* < 0.001). BEX3, in comparison, was overexpressed in the primary tumor. BEX4 expression in OSCC cell lines was re-activated after zebularine and TSA treatment. High BEX4 expression could suppress proliferation of OSCC in vitro. Subcutaneous tumor volume of BEX4-overexpressing CAL27 was remarkably reduced in nude mice. Microarray experiment showed that S100A family members (S100A7, S100A7A, S100A8, S100A9 & S100A12) might be the downstream targets of BEX4 in OSCC.

**Conclusions:**

BEX4 functions as tumor suppressor by inhibiting proliferation and growth of oral cancer. Decreased BEX4 contributes to the increased proliferative propensity of OSCC.

## Background

Epithelial cancer derived from the oral mucosa is a common head and neck cancer [1]. Histologically, oral squamous cell carcinoma (OSCC) is the major subtype contributing to over 90 % of the cases [2]. Despite the advances in cancer detection techniques and treatment methods in the past decade, the incidence of OSCC remains high and the 5-year survival rate of OSCC patients remains unsatisfactory [3]. OSCC is an aggressive head and neck cancer due to the rapid proliferative capability and high invasive nature [4]. The survival rate of OSCC patients showed close association with the tumor size [5]. It has been shown that tumor size is an independent predictive factor for survival of OSCC patients [6]. Therefore, a better understanding on the underlying mechanisms involve in OSCC growth is important to improve the prognosis of OSCC patients.

Silencing key tumor suppressor genes by epigenetic mechanisms are essential for the carcinogenic development of OSCC. Identifying key methylated genes with functional implications in the development of OSCC would be useful in diagnosis and as therapeutic targets [7]. High-throughput parallel screening using expression and methylation microarrays had shown that brain-expressed X-linked (BEX) members were susceptible to epigenetic inactivation in OSCC [8]. Human BEX members (BEX1, BEX2, BEX3, BEX4, BEX5) are all located on chromosome Xq22. They are highly conserved genes among different species (including chimp, mouse, rat, dog and human) implying their importance in cellular functions [9]. At present, little is known about the functional implications of BEX family members in the progression of human malignancies. It has been reported that BEX family members could regulate cell cycle and apoptotic signaling [10, 11].

BEX family members displayed distinct tissue distribution and have varying roles dependent on different cellular context. Thus, in the present study, we first explored the expression patterns of the human BEX family members in OSCC. Given that BEX4 expression was remarkably reduced in our cohort, we hypothesized that BEX4 could possibly play a tumor-suppressing role in OSCC. Further, whether DNA methylation or chromatin remodeling would be a possible cause of BEX4 suppression in OSCC will also be investigated. The functional role of BEX4 on tumor growth was examined using OSCC cell lines and xenograft models. As there was no information about the functional role of BEX4 in human malignancies, global gene expression change of BEX4-overexpressing OSCC cell line will be explored using microarray.

## Methods

### Cell cultures

Human tongue squamous cell carcinoma cell line CAL27 was obtained from American Type Culture Collection. Buccal squamous cell carcinoma cell line YD-38 was obtained from Korean cell line bank. Both cell lines were maintained in RPMI 1640 medium supplemented with 10 % fetal bovine serum, 100 U/ml penicillin and 100 μg/ml streptomycin. Drug treatment started after cell seeding for 24 h. For zebularine treatment, the cells were harvested after 72 h. For trichostatin A (TSA) treatment, the cells were harvested after 24 h.

### Clinical samples

Sixty-five OSCC and the paired normal tissues were examined. Tissue were obtained from patients underwent surgery in Division of Head and Neck, Queen Mary Hospital, Hong Kong. All the cancer tissues were squamous cell carcinoma. Consent of tissue donation for research purpose was obtained before sample collection. The study was approved by the ethnic review board of our institute (registered number: UW12-123).

### RNA extraction and real-time quantitative polymerase chain reaction (qPCR)

Total RNA was extracted using Trizol reagent (Invitrogen). First-strand cDNA synthesis was performed using High Capacity cDNA Reverse Transciption Kit (ABI). Transcript level was evaluated by qPCR using FastStart Universal Probe Master (Roche Applied Science) on LightCycler® 480 (Roche Applied Science). Table 1 listed the primers and probes used in this study. Reactions were performed at 95 °C for 10 min followed by 45 cycles of 95 °C for 15 s and 60 °C for 1 min. Expression levels were evaluated using the comparative threshold cycle methods.

**Table 1:** Primers and probes for qPCR

Name	Forward primer (5’-3’)	Reverse primer (5’-3’)	Probe number^a^
BEX1	CACCTCGTGGCGAGAATC	CTCTTTGGACTCCATTACTCCTG	63
BEX2	TCCTGAGGCTACGACCTTTC	CGATTCTCGACGTGAGGTG	16
BEX3	CTTCGGTGCAGTCGTCACT	ACACTTAGCCTCGCAGACCT	24
BEX4	GCCCCGAAATTAGGAAGC	TGTTTGCCGCTAGTTCCTCT	15
BEX5	GCCGATTTCAAGGCTAAGAG	GGACATTTTCCATGTTGAGTTTTT	34
RSAD2	CACGTAAACTAGATCAGGGAACAA	TCAACAAACTGCATGGGATATT	55
S100A9	CTCCCACGAGAAGATGCAC	GAGGCCTGGCTTATGGTG	81
IFI6	AACCGTTTACTCGCTGCTGT	GGGCTCCGTCACTAGACCTT	40
PTPRZ1	GGCCTGTTGTCGTCCACT	TCTGCTGCAACATACTGTCTAGC	67
MS4A4A	GGAATTCTGCATTGCTGTGTC	TGATGGCAGAATTAACACAACC	13
FABP5	GAGTGGGATGGGAAGGAAAG	GATCCGAGTACAGGTGACATTG	22
S100A7	CCAAACACACACATCTCACTCA	TCAGCTTGAGTGTTGCTCATC	33
S100A8	GCCAAGCCTAACCGCTATAA	ATGATGCCCACGGACTTG	34
SPRR1B	CAGAGTATTCCTCTCTTCACACCA	CAAGGCTGTTTCACCTGCT	3
S100A12	CACATTCCTGTGCATTGAGG	GGTGTCAAAATGCCCCTTC	31
S100A7A	CATAGCCGCAGACTACCACA	ACTGGCTTCCCCCAGAAC	21
SPRR1A	AGCCCAAGGTTCCAGAGC	TTCTGCTTGGTCTTCTGCTG	67
IFI44	AGCCTGTGAGGTCCAAGCTA	GAACATCCTTTACAGGGTCCAG	6
PGLYRP3	CCCCACTCCCTTTGAGACT	CTTGGCTGGTGAGGGTTG	3
IL1R1	GTCTCTGCTCTTGCCTCGAC	TCAGCTAGAGAAAATGCGAGAA	63
CBX5	AGAAGATGAAGGAGGGTGAAAA	GCGATATCATTGCTCTGCTCT	7
PTX3	TGTATGTGAATTTGGACAACGAA	CATTCCGAGTGCTCCTGAC	58
PADI3	CAGCAATGACCTCAACGACA	GAGGTAGAGCACCGCATAGG	19
ANKRD22	CAGTGCCTTTGTTTCATTGG	CACCTGTCAGTGCTTTTCCA	74
LIF	TGCCAATGCCCTCTTTATTC	GTCCAGGTTGTTGGGGAAC	26
TMEM156	CATGGAATACCCGAATGATTG	CCAAGTGATCTTCATGGAACAA	80
GPRC5B	CCGCAGAGATGTGACTCG	GATGCCACGAACATTCGAC	78
FTH1	GCCAGAACTACCACCAGGAC	CATCATCGCGGTCAAAGTAG	1
SLC6A15	AAAGTGGGGGCTTAAACTGG	CATAGCCAAGCAAACCATGA	27
SLITRK6	GAAGAAAAAGAAACTCAGGGATCA	GGACAGACTAAATTGCCTGAAGA	7
PLLP	CACCCGGCCTTATAACCAG	AAGGCACTCACTCCATAGGC	9

^a^Universal ProbeLibrary

### Immunocytochemistry

OSCC cells were seeded on chamber slides and treated with zebularine and TSA. Cells were fixed with 4 % paraformaldehyde and permeabilized using 0.2 % Triton X-100 in PBS. After blocking, cells were incubated with anti-BEX4 antibodies (Abcam) and CF488-conjugated secondary antibodies (Biotium). Cells were visualized by fluorescent microscope (Nikon).

### Immunohistochemistry

Tissue samples embedded into paraffin blocks were cut into 4-μm sections. After deparaffinization and rehydration, sections were microwaved in 10 mM sodium citrate buffer for antigen retrieval and stained with anti-BEX4 antibodies (Abcam) at room temperature for 1 h. DAKO EnVision + System, HRP (DAKO) was used for visualization of immunoreaction. Then, sections were stained with Mayer’s hematoxylin, dehydrated and photographed under light microscope. BEX4 protein expression level was graded as low, moderate or high depending on the staining intensity. Association between BEX4 expression and the clinicopathological parameters of OSCC patients was evaluated by Chi-square test or Fisher’s exact test.

### Lentivirus vector construction, lentivirus production and infection

Full-length BEX4 coding sequence was cloned into lentiviral expression vector pCDH (System Biosciences) to generate the BEX4-expressing vector (pCDH-BEX4 vector). Vector sequences were verified by direct sequencing. Virus packaging was performed by transient transfection into 293 T cells using Lipofectamine® 2000 Transfection Reagent (Invitrogen). OSCC cell lines CAL27 and YD-38 were transduced with medium containing lentivirus.

### SiRNA transfection

BEX4 siRNA-1, BEX4 siRNA-2 and negative control siRNA (QIAGEN) were transfected into OSCC cells using HiPerFect transfection reagent (QIAGEN) according to the manufacturer’s protocol. After 72 h, cells were collected and the efficiency of BEX4 silencing was determined by qPCR and immunocytochemistry.

### Western blot

His-tag BEX4 protein was obtained from Novus Biologicals. Protein was extracted from cells using RIPA lysis buffer. Protein concentration was measured by BCA protein assay system (Pierce). Proteins separated by sodium dodecyl sulphate-poly-acrylamide gel electrophoresis were transferred to PVDF membranes and incubated with anti-BEX4 antibodies (Abcam). After incubation with horseradish peroxidase-conjugated goat anti-rabbit secondary antibody, hybridization signals were developed using ECL Plus Western Blotting Detection Reagents (Amersham Biosciences).

### Real-time proliferation assay

Real-Time cell kinetic analyzer xCELLigence RTCA (ACEA Biosciences). was used to monitor the dynamic changes of cell proliferation. Data analysis was performed using RTCA Control Unit and the preinstalled RTCA software. For real-time proliferation assay, E-plate 16 was used. Cells were seeded directly onto E-plate. Changes in baseline impedance resulting from the increase of cell numbers were monitored by gold micro-electrodes located at the bottom of E-plate. The proportional changes in impedance were recorded continuously and expressed as cell index (CI). Change of CI with time was monitored continuously for 72 h.

### Cell cycle analysis

Apoptosis was determined by propidium iodide (PI) staining. Cells were harvested and fixed with 70 % ethanol for 24 h at 4 °C. The cells were stained with PI and RNase A in PBS for 30 min in the dark. Cell-cycle distribution was analyzed with a FACS flow cytometer (BD Biosciences).

### Animal models

Athymic nu/nu mice (5 weeks old, weight range: 18–22 g) were used. CAL27 with pCDH-BEX4 vector or empty vector pCDH were injected subcutaneously into the right flank of mice. Four animals were used in each group. Tumor growth was measured daily with calipers in two dimensions. After 30 days, mice were euthanized. The animals were maintained under pathogen-free conditions with controlled temperature and humidity.

### Microarray

Affymetrix whole-transcript expression microarray Human Exon 1.0 ST was used. All the RNA samples were tested with Agilent 2100 Bioanalyser (Agilent Technologies). All the samples RNA had RIN value over 8. All the tests were performed in the Centre for Genomic Sciences, the University of Hong Kong. Data analysis was evaluated using GeneSpring version 13 (Agilent Technologies). Genes with more than 1.5-fold difference and p value below 0.01 were selected for qPCR validation.

### Statistical analysis

Data were expressed as mean ± standard deviation. *T*-test or Chi square test were used to compare difference between groups as appropriate. Data analysis was performed using SPSS® for Windows version 14.0 (SPSS, Inc., Chicago, IL, USA). All the tests were two-sided. P-values <0.05 were considered as statistically significant. SurvExpress (Interface v2.0, Database Update: Sep 30, 2015) was used to perform survival analysis on oral cancer dataset GSE26549 as described [12]. The dataset contains global gene expression data from 86 oral leukoplakia tissue samples examined with Affymetrix Human Gene 1.0 ST Array [13].

## Results

### Aberrant expression of BEX4 in oral cancers

**Fig. 1:**
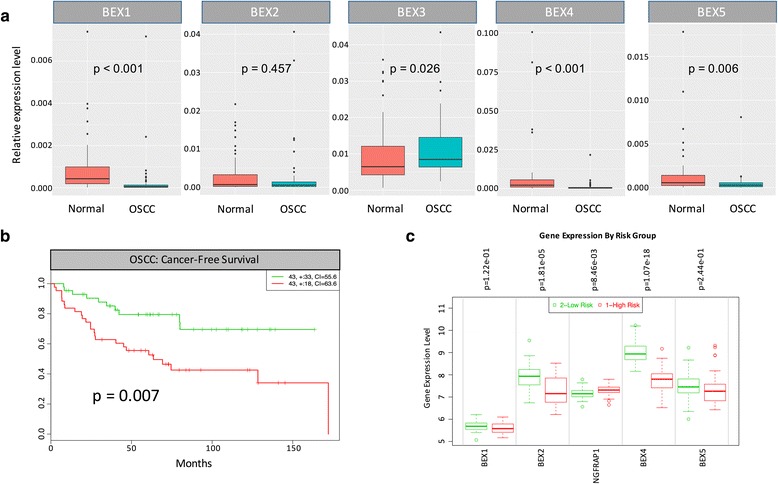
Expression of BEX family members in OSCC. **a** Boxplots showing expression level of BEX family members in OSCC and paired normal tissues. Each sample were normalized to GAPDH and displayed using 2^-ΔCt^ values. **b** Cox survival analysis of cancer-free survival was performed on oral leukoplakia patients based on the BEX4 expression expression level. **c** Boxplots showing the expression level of BEX family members in different risk group

To identify the key BEX family members involved in OSCC, we used qPCR to quantify BEX1-5 transcript levels in 65 primary OSCC tissues and compared with the paired normal epithelia. In comparison with the normal counterpart, expression of BEX1 (*p* < 0.001), BEX4 (*p* < 0.001) and BEX5 (*p* = 0.006) were significantly reduced in the OSCC tissues (Fig. 1a). In contrast, BEX3 expression showed significant elevation in OSCC (*p* = 0.026). For BEX2, no significant difference was observed between the OSCC and paired normal tissues (*p* = 0.457). The characteristics of the 65 patients examined were shown in Table 2.

**Table 2:** Association of BEX4 mRNA expression level with the clinicopathological variables in 65 patients with OSCC

Variables	Cases	BEX4 expression		*P* value*
		Low (%)	High (%)	
Gender	-	-	-	0.875
Male	40	20 (50)	20 (50)	-
Female	25	13 (52)	12 (48)	-
Age	-	-	-	0.708
> 59	32	17 (53)	15 (47)	-
≤ 59	33	16 (48)	17 (52)	-
T-stage	-	-	-	0.174
T1-2	29	12 (41)	17 (59)	-
T3-4	36	21 (58)	15 (42)	-
Nodal stage	-	-	-	0.694
Negative	37	18 (49)	19 (51)	-
Positive	28	15 (54)	13 (46)	-

*Pearson chi-square test

### Low BEX4 expression was associated with poor outcome in patients with oral pre-neoplastic lesions

We analyzed the association between BEX family member expression and the outcome of 86 oral leukoplakia patients from GSE26549 using SurvExpress. Low BEX4 expression was associated with poor cancer-free survival of oral leukoplakia patients (Hazard ratio = 2.76, *p* = 0.0072) (Fig. 1b, c). The correlation of low BEX4 expression with poor cancer-free survival in leukoplakia patients was more significant than other BEX family members including BEX1, BEX2, NGFRAP1 (BEX3) and BEX5 (Fig. 1c).

### Low BEX4 expression was associated with advanced tumor stage

**Fig. 2:**
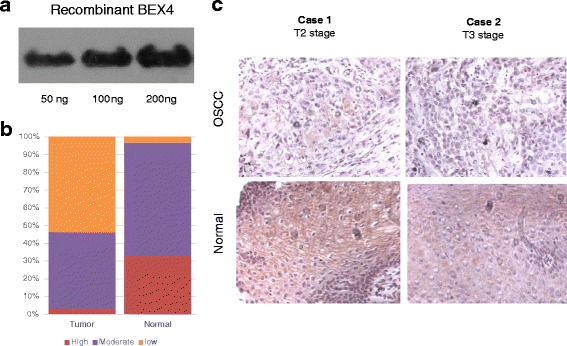
Immunohistochemical analysis of BEX4 expression in OSCC. In total, 30 paired tumor and normal tissues from patients with OSCC. **a** Specificity of BEX4 antibody. Western bot analysis showed that the anti-human BEX4 antibody can specifically detect the recombinant human BEX4 protein. **b** Distribution of tissue BEX4 expression level in our OSCC cohort. **c** Representative immunohistochemical staining images showing BEX4 expression on OSCC tissues and paired normal epithelia

To confirm the specificity of BEX4 antibody, Western blot was performed on the his-tagged recombinant human BEX4 protein. Signal of the immunoreaction increased in parallel with the concentration of recombinant BEX4 (Fig. 2a). In tissues obtained from OSCC patients, majority of normal tissues (96.9 %) showed moderate or high expression of BEX4. In the OSCC tissues, low expression of BEX4 was observed in 53.3 % tumor samples from patients with OSCC, while it was only found in 3.3 % paired normal tissues (Fig. 2b). Moreover, 75 % tumor tissues with T3-4 stage exhibited low BEX4 expression. In contrast, only 10 % tumor tissues with T1-2 stage displayed low BEX4 expression (Table 3). Low BEX4 level was associated with advanced stage tumor (*p* = 0.001, Table 3). Representative images of BEX4 staining on oral tissues were shown in Fig. 2c.

**Table 3:** Association of BEX4 protein expression level with the clinicopathological variables in 30 patients with OSCC

Variables	Cases	BEX4 expression		*P* value
		Low (%)	Moderate + High (%)	
Gender	-	-	-	0.796^a^
Male	20	11 (55)	9 (45)	-
Female	10	5 (50)	5 (50)	-
Age	-	-	-	0.464^a^
≤54	15	9 (60)	6 (40)	-
>54	15	7 (47)	8 (53)	-
T-stage	-	-	-	**0.001** ^**b**^
T1-2	10	1 (10)	9 (90)	-
T3-4	20	15 (75)	5 (25)	-
Nodal stage	-	-	-	0.464^a^
Negative	15	7 (47)	8 (53)	-
Positive	15	9 (60)	6 (40)	-

^a^Pearson chi-square test; ^b^Fisher’s exact test; Bold data indicated statistically significant

### BEX4 was epigenetically silenced in OSCC

**Fig. 3:**
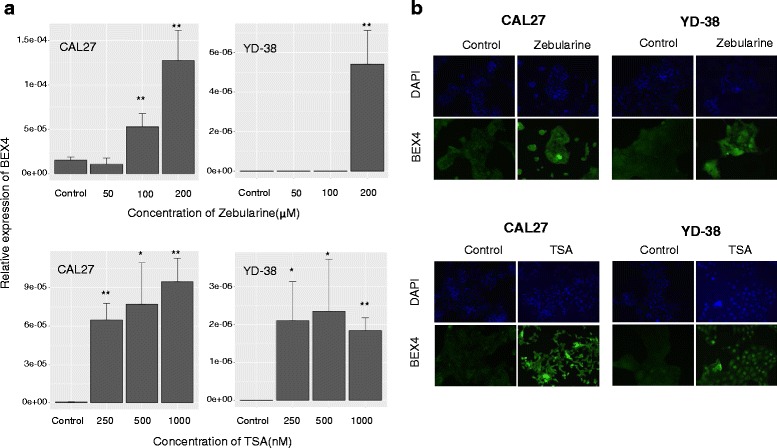
Epigenetic drugs induced BEX4 expression in OSCC. **a** Zebularine and TSA treatment upregulated BEX4 expression in OSCC cell lines. **b** Immunostaining analysis showed that BEX4 protein increased in CAL27 and YD-38 after zebularine and TSA treatment

Next, we sought to examine whether DNA methylation and histone acetylation are the major epigenetic alterations leading to BEX4 suppression in OSCC. DNA methylation was controlled by DNA methyltransferases that catalyze the addition of a methyl group to DNA. Zebularine inhibited DNA methyltransferases through the formation of a covalent complex with them [14]. Histone deacetylases (HDACs) catalyze the removal of acetyl groups of histone, leading to chromatin condensation and transcriptional suppression. TSA was a specific inhibitor of HDAC activity [15]. Following treatment with zebularine and TSA, dose-depending increase in BEX4 expression was observed in CAL27 cells (Fig. 3). The protein expression level of BEX4 was also enhanced following zebularine and TSA treatment (Fig. 3).

### BEX4 reduced the proliferation and tumor growth of OSCC

**Fig. 4:**
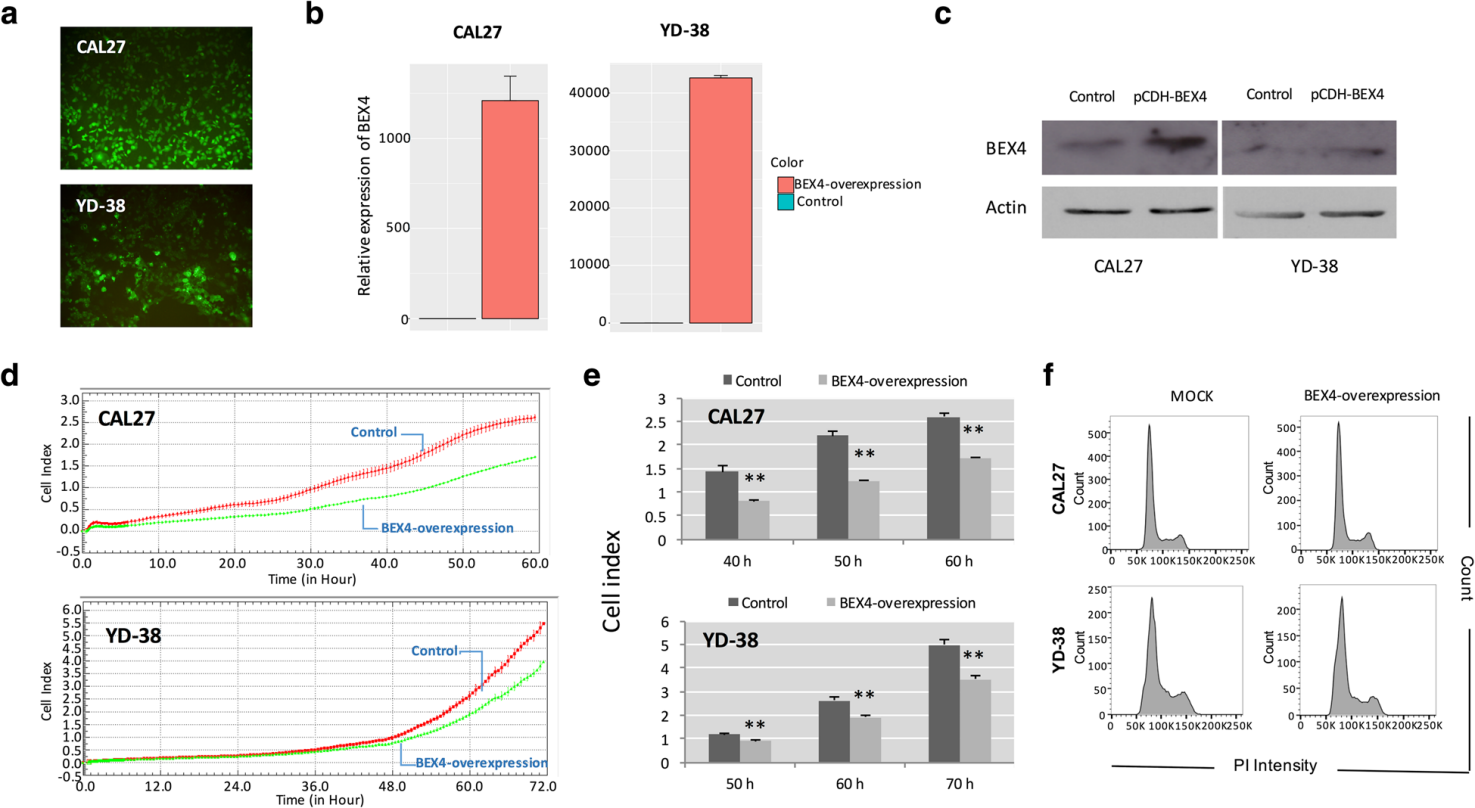
Effect of BEX4 overexpression on OSCC proliferation. **a** pCDH-BEX4 containing OSCC cells showed positive GFP signals. **b** QPCR analysis showed remarkable increase in BEX4 transcript levels. **c** Western blot analysis showed increase in protein expression in the infected cells. **d** Upregulated BEX4 expression in OSCC inhibited cell proliferation. **e** Time-dependent reduction in proliferation capacity of the BEX4-overexpressed OSCC. **f** Flow cytometry analysis with PI staining showed that BEX4 expression did not induce apoptosis in the OSCC cells

**Fig. 5:**
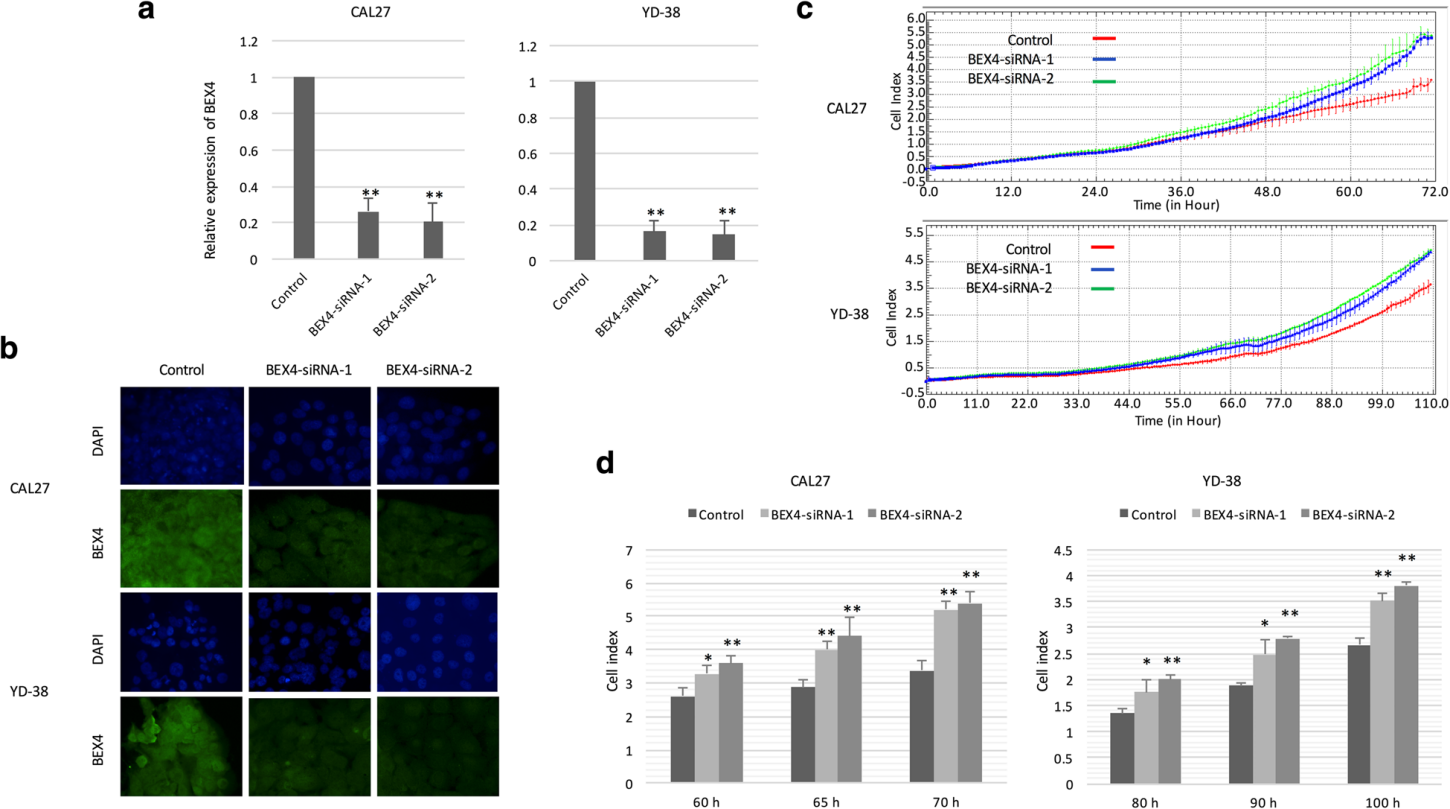
Effect of BEX4 knockdown on OSCC proliferation. **a** OSCC transfected with 2 different BEX4-specific siRNAs showed significant reduction in endogenous BEX4 transcript levels. **b** Immunostaining indicated BEX4 protein reduction in OSCC transfected with BEX4 siRNAs. **c** Knockdown BEX4 with siRNA promoted OSCC proliferation. **d** Time-dependent increase in proliferation capacity of BEX4 knockdown OSCC

Having established that BEX4 expression was suppressed in OSCC, we hypothesized that BEX4 has a tumor suppressing function in OSCC. We generated BEX4-overexpressing OSCC cell lines (Fig. 4a–c) and measured the change of proliferation rate. BEX4-overexpressing cells showed significant reduction in OSCC proliferation (Fig. 4d, e). Apoptotic cells were determined by PI staining using flow cytometry. Flow cytometry analysis showed that BEX4 overexpression in OSCC did not induce apoptosis of OSCC (Fig. 4f). To confirm our results, two different BEX4-specific siRNAs were used to silence the expression of BEX4 in OSCC cells (Fig. 5a, b). Silence of BEX4 resulted in a significant increase in the proliferation of CAL27 and YD-38 cells (Fig. 5c, d).

**Fig. 6:**
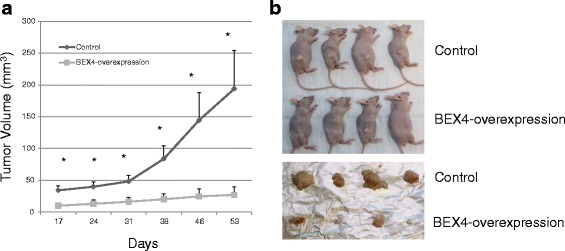
BEX4 inhibited growth of CAL27 xenograft. **a** Xenograft growth curve. **b** Xenograft excised from the indicated time point

In the animal model, BEX4-overexpressing xenograft developed from CAL27 cells had a significantly smaller tumor volume and slower growth rate (Fig. 6). Tumor volume of control xenograft increased by 4.7-fold from day 17 to day 53. In contrast, tumor volume of BEX4-overexpressing xenograft increased by only 1.7-fold (Fig. 6). The data is consistent with the cell line models suggesting that BEX4 was involved in controlling OSCC growth.

### S100A family is the potential downstream targets regulated by BEX4

**Fig. 7:**
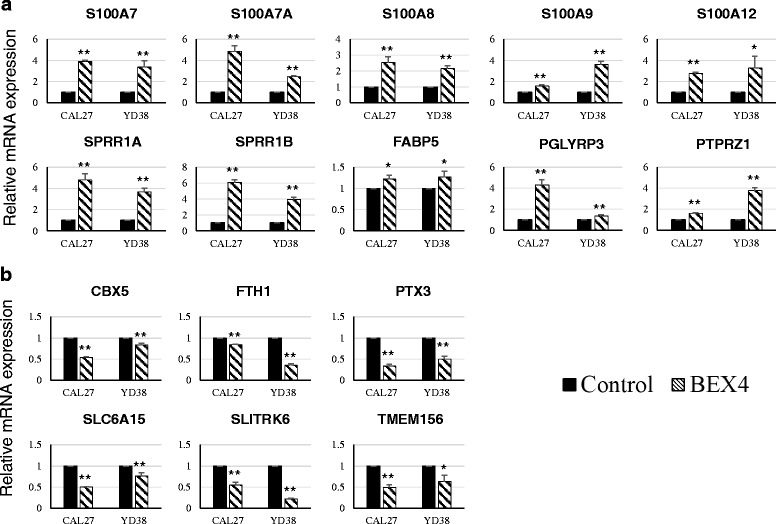
Gene expression changes in BEX4-overexpressing CAL27. Global gene expression changes were evaluated using Affymetrix Human Exon 1.0 ST. The differential expressed genes were further evaluated in BEX4-overexpressing OSCC cell lines. **a** Gene up-regulated in BEX4-overexpressing CAL27 and YD-38. **b** Gene down-regulated in BEX4-overexpressing CAL27 and YD-38

As there is no sufficient information about the regulatory mechanisms of BEX4 in human diseases, we used microarray to explore the global gene expression changes in BEX4-overexpressing OSCC cell line CAL27. Twenty-six genes showed differential expression (Table 4). The filtered genes were then validated in BEX4-overexpressing Cal27 and YD-38 again. Of which, the expression patterns of 16 genes were consistent with the microarray results (Fig. 7). According to the expression patterns in qPCR results, we classified the genes into BEX4-suppressed genes (S100A7, S100A8, S100A7A, RSAD2, S100A12, SPRR1B, S100A9, SPRR1A, MS4A4A, IF16, PGLYRP3, IL1R1, IFI44, FABP5, PTPRZ1) and BEX4-induced genes (FTH1, PLLP, SLC6A15, PTX3, CBX5, TMEM156, GPRC5B, ANKRD22, LIF, PADI3, SLITRK6). Among the differentially expressed genes, expression changes of multiple S100A family members were observed in BEX4-overexpressing Cal27 and YD-38. S100A7, S100A7A, S100A8, S100A9 and S100A12 were remarkably increased in the BEX4-overexpressing OSCC cell lines.

**Table 4:** Gene expression profile of BEX4-overexpressing CAL27

Transcripts cluster Id	Gene symbol	Regulation	Fold-change	Gene name
2436051	S100A7	Up	3.03	S100 Calcium Binding Protein A7
2435989	S100A8	Up	2.80	S100 Calcium Binding Protein A8
2359691	S100A7A	Up	2.47	S100 Calcium Binding Protein A7A
2468351	RSAD2	Up	2.00	Radical S-Adenosyl Methionine Domain Containing 2
2435981	S100A12	Up	1.79	S100 Calcium Binding Protein A12
2359521	SPRR1B	Up	1.79	Small Proline-Rich Protein 1B
2359664	S100A9	Up	1.72	S100 Calcium Binding Protein A9
2359492	SPRR1A	Up	1.68	Small Proline-Rich Protein 1A
3332298	MS4A4A	Up	1.65	Membrane-Spanning 4-Domains, Subfamily A, Member 4A
2403261	IFI6	Up	1.64	Interferon, Alpha-Inducible Protein 6
2435949	PGLYRP3	Up	1.59	Peptidoglycan Recognition Protein 3
2496962	IL1R1	Up	1.58	Interleukin 1 Receptor, Type I
2343511	IFI44	Up	1.56	Interferon-Induced Protein 44
3104933	FABP5	Up	1.54	Fatty Acid Binding Protein 5 (Psoriasis-Associated)
3021377	PTPRZ1	Up	1.52	Protein Tyrosine Phosphatase, Receptor-Type, Z Polypeptide 1
4037708	FTH1	Down	2.39	Ferritin, Heavy Polypeptide 1
3693141	PLLP	Down	1.67	Plasmolipin
3464276	SLC6A15	Down	1.61	Solute Carrier Family 6 (Neutral Amino Acid Transporter), Member 15
2649367	PTX3	Down	1.60	Pentraxin 3, Long
3456630	CBX5	Down	1.59	Chromobox Homolog 5
2766289	TMEM156	Down	1.54	Transmembrane Protein 156
3683377	GPRC5B	Down	1.54	G Protein-Coupled Receptor, Class C, Group 5, Member B
3299469	ANKRD22	Down	1.54	Ankyrin Repeat Domain 22
3957160	LIF	Down	1.54	Leukemia Inhibitory Factor
2322818	PADI3	Down	1.52	Peptidyl Arginine Deiminase, Type III
3519840	SLITRK6	Down	1.51	SLIT And NTRK-Like Family, Member 6

## Discussion

Our results indicated that BEX4 functions as tumor suppressor in controlling proliferation and growth of OSCC. At present, little is known about the expression status and functions of brain expressed X-linked family in OSCC. Thus, we first examined the mRNA levels of BEX1, BEX2, BEX3, BEX4 and BEX5 in our OSCC tissues and compared with the paired normal epithelia. We identified that BEX4 suppression is a common event in our OSCC and is potentially associated with the poor outcome of patients. The association between low BEX4 with poor outcome of patients with oral pre-neoplastic lesions suggested that reduced BEX4 is an early event in the development of OSCC.

OSCC is a male dominant disease. Recent epidemiology study revealed that there is an increasing number of female OSCC cases [16]. In our cohort, reduced BEX4 expression is also observed in the female group. Functional analysis revealed that BEX4 reduced proliferation propensity of OSCC. The remarkable reduction of xenograft volume further supports our suggestion that BEX4 controls the proliferative growth process of OSCC. Taken together, our results provide evidence showing a putative tumor-suppressing functions of BEX4 in OSCC.

Members of BEX family have been implicated in regulating apoptosis in various human cancer cells and normal cells. In breast cancer, BEX2 could modulate ceramide-induced apoptosis via protein phosphatase 2A [17, 18]. BEX1 interacted with B-cell lymphoma 2 (BCL-2) and inhibited the formation of BCL-2/BCL-2-associated X protein (BAX) complex, leading to enhancement of imatinib-induced apoptosis in human leukemic cell line K562 [19]. BEX3 could interact with the death domain of p75 neurotrophin receptor (p75NTR) and induce apoptosis upon nerve growth factor (NGF) treatment in HEK293 cells [20]. In light of the regulating role of BEX family in apoptosis, we investigated whether BEX4 overexpression affect apoptosis in OSCC. Unlike other BEX family proteins, BEX4 had no inducing effect on apoptosis and cell cycle arrest.

At present, the functional roles of BEX family members in cancer remain unclear. In OSCC, it has been reported that BEX1 is involved in modulating nuclear factor-κB (NF-κB) signaling pathway [8]. BEX2 expression could possibly be modulated by the aberrantly activated mechanistic target of rapamycin (mTOR) in cancer cells [21]. BEX protein family is also known as intrinsically disordered proteins (IDPs), which exist as dynamic ensemble of conformation with no discrete 3D structure. IDPs are suggested to play an important role in tumorigenesis and are involved in modulating multiple gene regulating signaling pathways by their ability to recognize multiple interaction partners [22]. Our results revealed that BEX family member, BEX4, is one of the IDP involved in the pathogenesis of OSCC.

Epigenetic silencing of tumor suppressor gene is a characteristic feature of OSCC. Existing data suggests that DNA methylation and chromatin remodeling play an important role in modulating BEX family expression in human malignancies [23, 24]. In glioma, BEX1 and BEX2 are silenced and the expression could be activated by treatment with DNA methyltransferase inhibitor and histone deacetylase inhibitor [25]. Our results are consistent with the current knowledge suggesting that BEX4 is epigenetically silenced in OSCC.

OSCC overexpressing BEX4 showed significant up-regulation of S100A family members (S100A7, S100A8, S100A9, S100A12, S100A15 or S100A7A). S100A is a family of calcium binding protein involved in multiple processes of cancer development [26]. Of which, S100A7 dysregulation is believed to be involved in a wide range of pathological processes associated with OSCC. S100A7 (also known as psoriasin) was expressed in early stage oral cancer. S100A7 expression was reduced in tumor in late stage [27]. Additionally, highly differentiated OSCC usually had high expression of S100A7 as compared to the moderate or poorly differentiated counterparts [28]. Forced expression of S100A7 is accompanied by repressed cell proliferation and tumor progression in orthotopic tongue tumor model [27].

Apart from S100A7, aberrant expression of other S100A family members is emerging as an important event in head and neck cancers. In head and neck squamous cell carcinoma, S100A8/A9 (calprotectin) could suppress cell growth by inducing G2/M cell cycle checkpoint arrest [29]. The mRNA and protein expression levels of S100A8/A9 are decreased in head and neck cancers. S100A8/A9 can modulate G2/M Cdc2/cyclin B1 complex activation resulting in G2/M checkpoint arrest [29]. For S100A12, high level in tumor tissue is a good prognostic indicator for oropharyngeal squamous cell carcinoma patient [30]. S100A7A (koebnerisin) are highly homologous to S100A7. Both S100A7A and S100A7 could serve as oncogenic inactivator in human malignancies [31].

The molecular mechanisms underlying S100A proteins dysregulation remains unclear. In OSCC, it has been shown that overexpression of β-catenin decreased the expression of S100A7, while silence of β-catenin enhanced its expression, indicating that β-catenin signaling negatively modulated S100A7 [27]. In addition, c-Myc suppressed S100A7 expression by directly binding to its promoter [32].

## Conclusions

Based on the current results, we suggested that BEX4 controls OSCC proliferation. Although the mechanisms underlying BEX4 suppression in OSCC remains unresolved, induced expression of BEX4 in the presence of epigenetic drugs suggested that BEX4 is epigenetically silenced in OSCC. Taken together, we suggested that BEX4 functions as a novel tumor suppressor gene involved in OSCC.

## Abbreviations

BAX: BCL-2-associated X protein
BCL-2: B-cell lymphoma 2
BEX: brain-expressed X-linked
HDACs: histone deacetylases
HNSCC: head and neck squamous cell carcinoma
mTOR: mechanistic target of rapamycin
NGF: nerve growth factor
OSCC: oral squamous cell carcinoma
p75NTR: p75 neurotrophin receptor
TSA: trichostatin A
